# The lived experiences of caregivers of people with epilepsy in Botswana

**DOI:** 10.4102/phcfm.v18i1.5534

**Published:** 2026-09-04

**Authors:** Jappy M. Toe, Tebogo T. Mamalelala, Esther I. Ntsayagae

**Affiliations:** 1 School of Nursing, Faculty of Medicine and Health Science, University of Botswana, Gaborone, Botswana; 2 Partners in Health, Maryland County, Liberia

**Keywords:** caregiver stress, epilepsy caregiving, emotional and financial impact, psychosocial support, stigma

## Abstract

**Background:**

Epilepsy is a common neurological disorder affecting about 50 million people worldwide. Caregivers of people with epilepsy face significant emotional, physical and psychological distress due to the demands of caregiving. Limited resources, stigma and the lack of a proper support system often worsen these challenges.

**Aim:**

This study aimed to explore the lived experiences of caregivers of individuals with epilepsy in Botswana.

**Setting:**

This study was conducted in Gaborone, the capital city of Botswana, located in Botswana’s South-East District.

**Methods:**

An interpretive phenomenological approach was used to capture the lived experiences of 13 caregivers, recruited through purposive sampling. In-depth interviews were conducted to gather qualitative data, which were analysed using the General Inductive Approach.

**Results:**

Caregivers reported significant emotional distress, including anxiety, stress and social isolation due to the unpredictable nature of seizures. Financial difficulties were dominant concern, with many caregivers losing employment status due to caregiving responsibility. High cost of medication and access to healthcare services was another burden while stigma surrounding epilepsy further exacerbated emotional strain. Additionally, caregivers reported limited coping strategies, which further contributed to their psychological distress.

**Conclusion:**

The study highlights the urgent need for culturally sensitive interventions to address the psychological and economic burden faced by caregivers. Strengthening support system, ensuring reliable access to medication and providing financial aid could significantly improve caregivers’ well-being and by extension, the quality of life for individuals living with epilepsy in Botswana.

**Contribution:**

This study offers important insights into caregiver experiences in epilepsy, emphasising the need for family inclusive, caregiver centred nursing practices, educational enhancement and improved community policies in Botswana. It highlights the importance of structured psychosocial support, integrating epilepsy care into home services, and culturally relevant coping strategies. These findings aim to guide targeted interventions and create more responsive health systems in similar resource-limited settings.

## Introduction

Epilepsy is one of the most prevalent neurological conditions worldwide and present substantial challenges not only for individuals living with the condition, but also for their families and caregivers.^1^ Beyond seizure control, epilepsy exerts profound psychosocial, economic and emotional effects on caregivers, often compromising their quality of life and increasing their vulnerability to psychological distress.^1^ These challenges are particularly pronounced in low- and middle-income countries (LMICs), including many African settings, where cultural beliefs surrounding epilepsy, limited access to healthcare services and economic constrains intensify the caregiving experience.

Evidence from LMICs consistently demonstrates the multidimensional burden associated with epilepsy caregiving. In Uganda, caregivers experience substantial financial strain due to medical expenses and income loss resulting from disrupted employment.^2^ Similarly, studies from South Africa report high unemployment rates among caregivers of people with epilepsy, reflecting the competing demands of caregiving and economic participation.^3^ In the Democratic Republic of Congo, epilepsy management has been shown to consume nearly half of household income, compounded by reliance on costly traditional treatment.^4^ Comparable findings have been reported in Nigeria, where female caregivers in particular experience high unemployment, low educational attainment and elevated levels of anxiety and depression.^5^ Collectively, these studies indicate that epilepsy caregiving extends beyond clinical management to encompass interrelated economic, psychological and social stressors that shape caregivers daily functioning and well-being.

The psychological impact of caregiving for individuals with epilepsy is well documented. Caregivers frequently reported heightened stress, anxiety, sleep disturbances and depressive symptoms associated with the unpredictability of seizures, long-term caregiving responsibilities and concerns about safety and social stigma.^6^ Emotional burden of epilepsy can be as disabling as its clinical manifestations. During the coronavirus disease 2019 (COVID-19) pandemic, caregivers and people with epilepsy experienced increased psychological distress due to social isolation, reduced access to healthcare services and disruption in medication supply, underscoring the close interplay between medical conditions, mental health and social context.^7^

Social support has emerged as a critical protective factor in mitigating psychological distress among caregivers of individuals with epilepsy. Caregivers who receive consistent support from family members, peers and healthcare professionals report lower levels of anxiety and depression and improved emotional adjustment. Strong social networks have been shown to foster adaptive responses to caregiving demands, while support from healthcare providers through counselling and psychoeducation enhances caregivers’ resilience and coping capacity.^8,9,10,11^ These findings suggest that social support not only buffers psychological strain, but also influences caregiving behaviours and long-term adaption.

In addition to external support, caregivers’ psychological outcomes are shaped by the coping mechanism they employed. Research distinguishes between problem-focused coping, aimed at addressing practical challenges and emotional-focused coping, which involves regulating emotional responses to stress. Adaptive coping strategies such as emotional regulation, relaxation and self-compassion have been associated with reduced caregiver burden and improved psychological well-being.^12,13^ Conversely, maladaptive coping mechanism, including avoidance and denial, are linked to increased emotional distress and social isolation.^14^ Importantly, coping mechanism are influenced by broader contextual factors such as cultural norms, stigma, available resources and health system support.

Within Botswana, existing caregiving research largely mirrors patterns observed across other LMIC settings, confirming the persistence of financial strain, psychological distress and limited psychosocial support among caregivers of people with epilepsy and related conditions. Caregiving-related expenses have been shown to frequently exceed household income, contributing to sustained economic hardship,^15^ while epilepsy-focused studies report high levels of anxiety, depression, sleep disturbance, work disruption and time burden among caregivers.^16^

Evidence from broader neurological and mental health caregiving further highlights the influence of stigma, emotional exhaustion, social isolation and gaps in caregiver education and follow-up support within the health system.^17,18,19^ Collectively, these findings suggest that while caregiver burden in Botswana is well recognised, it remains insufficiently addressed through structured, caregiver-centred psychosocial interventions. Research on caregiving has primarily focused on human immunodeficiency virus (HIV), disabilities and mental illness, rather than epilepsy.^15,18,19,20^ As a result, the experiences of informal caregivers of individuals with epilepsy have not been thoroughly explored. This study aimed to explore the lived experiences of informal caregivers of individuals with epilepsy in Gaborone, Botswana.

## Research methods and design

### Study design

This study employed a qualitative research interpretive phenomenological design to explore the lived experiences of caregivers of individuals with epilepsy in Gaborone, Botswana. Interpretive phenomenology was appropriate because the study sought not only to describe caregiving experiences, but also to understand how caregivers made sense of those experiences within their social and caregiving context.^21,22,23,24^ This approach was well suited to capturing the emotional, psychological and social dimensions of epilepsy caregiving including caregiving challenges, coping strategies and support systems available to caregivers. Interpretive phenomenology goes beyond describing the essence of the phenomenon to examining meaning, context and the way experience is understood by those living it.^21,22,23,24^

### Setting

This study was conducted in Gaborone, the capital city of Botswana, located in Botswana’s South-East District. Princess Marina Hospital (PMH), Neurological Department at PMH, serves as the national referral centre for neurological care, including epilepsy. The Neurology Department at PMH offers a variety of specialised services, including an outpatient epilepsy clinic, inpatient care and multidisciplinary follow-up for both acute and chronic neurological conditions. The epilepsy clinic manages patients with new-onset seizures, refractory epilepsy and seizure-related comorbidities, receiving referrals from district and primary hospitals throughout Botswana. As a result, the clinic’s patient demographic includes both urban residents and those referred from rural and remote areas. During the study period, the department was staffed by a limited number of neurologists, along with medical officers, specialist nurses and allied health professionals such as physiotherapists and social workers, available upon referral.

### Study population

The targeted population for this study included informal caregivers of patients who received care at PMH, Neurological Department. Informal caregivers were defined as unpaid family members, relatives or close community members who provide day-to-day care for individuals living with epilepsy. Eligible participants included parents, siblings, spouses, relatives or guardians aged 18 years or older who had been actively involved in caregiving for at least 1 year.

### Sample size

In this study, the sample size was not predetermined; instead, it was determined by thematic data saturation.^25,26^ A sample of between 10 and 30 participants was expected.^27,28,29,30^ Thematic saturation was assessed concurrently with data collection through ongoing review of interview transcripts and emerging codes. Saturation was first observed after the 10th interview, when no new themes or substantive insights were identified. Data collection was subsequently extended to 13 participants to confirm saturation and ensure that no additional themes emerged.^30^

### Sampling

Purposive criterion sampling was used to select participants. Medical records at PMH were first reviewed to identify patients diagnosed with epilepsy, after which their primary informal caregivers were identified. Participants were deliberately selected based on predefined inclusion criteria, particularly caregiving duration, relationship to the patient and active involvement in daily care. Efforts were made to ensure variation in caregiving experiences by selecting caregivers from different communities within Gaborone and with diverse relational roles (e.g. parents, spouses, siblings). This approach ensured that the sample captured a broad range of perspectives relevant to the study objectives.

### Data collection

The interview guide was developed through a comprehensive review of pertinent literature on caregivers of individuals with epilepsy in Botswana. This process informed the formulation of open-ended questions specifically designed to elicit detailed narratives of caregivers’ experiences. The guide encompassed a variety of topics, including caregivers’ overall experiences, daily challenges encountered, emotional burdens, financial strains and the effects of caregiving on employment and personal time. Additionally, it addressed coping strategies, sources of support from family, friends and the healthcare system and caregivers’ self-care practices. The opening question of the interview is: ‘Can you describe your experience as a caregiver for someone with epilepsy?’ This question sets the stage for a conversation in which participants can share their personal stories and perspectives.

Interviews were conducted in the participants’ preferred language, either English or Setswana. All interviews were audio-recorded, anonymised, assigned unique numeric codes and transcribed verbatim by the principal researcher. Interviews conducted in Setswana were translated into English during transcription. To ensure accuracy, the audio recordings were consulted to resolve any inconsistencies, with input from the research assistant when necessary. The transcripts and translations were further verified by Tebogo T. Mamalelala and Esther I. Ntsayagae.

The interviews took place in participants’ homes to create a comfortable environment that encouraged open and authentic responses. Probing questions were used during the interviews to elicit deeper exploration of participants’ thoughts and feelings, thereby enhancing the richness of the collected data. All participants signed a consent form to participate in the research and were informed that their responses would be kept confidential. The interviews were recorded, and significant observations of the participants that could not be captured by audio recording were noted as field notes. Each interview lasted between 8 min and 38 min, with an average duration of 27 min.

### Data analysis

Data were analysed using Thomas’s General Inductive Analysis (GIA), a systemic approach that allows themes and patterns to emerge directly from participants’ narratives without imposing pre-existing theoretical frameworks.^31^ This method condenses raw qualitative data into a concise, organised format that remains closely linked to the study objectives and grounded in participants lived experiences.

All interviews were transcribed verbatim by Jappy M. Toe and checked by Tebogo T. Mamalelala, including pauses, non-verbal cues and emotional expression. Transcripts were checked against the original recording for accuracy. Using an inductive approach, open coding was applied by Jappy M. Toe, Tebogo T. Mamalelala and Esther I. Ntsayagae. to break transcripts into smaller segments. The codes were derived directly from the data rather than guided by prior assumptions.

Codes were compared and clustered into broader categories representing recurring ideas and shared experiences. Through an iterative process of refinement, these categories were reorganised and consolidated into themes that encapsulated the central issues expressed by caregivers. Themes were reviewed to ensure internal consistency and accurate representation of the data. Findings were presented narratively and supported by direct participants quotations to illustrate and validate the themes. Ensuring faithful representation of caregivers’ voice without pre-imposed theoretical constraints.

### Trustworthiness

Prolonged engagement, triangulation, member checking, peer briefing and persistent observation can strengthen credibility of the study.^30^ Credibility was also established through member checking with participating caregivers. After the interviews, preliminary themes and interpretations were shared with participants. Participants were asked to confirm whether the findings accurately reflected their experiences. Their feedback was used to refine the final themes.

Research assistants participated in a 2-day training session to establish credibility and consistency in data collection before the principal researcher began collecting data. The researcher provided detailed descriptions of participants’ backgrounds, caregivers’ settings and specific challenges faced by caregivers in Gaborone to enhance transferability.

To ensure dependability, the researcher had the study’s procedures evaluated by supervisors, maintaining transparency throughout the research process.^30^ Peers reviewed the clarity of the research methods and processes, while the research supervisor oversaw the study’s procedures. The researcher also ensured dependability by having the supervisor examine the collected data, its management and the data analysis process to confirm consistency.

Confirmability was established through reflexivity, acknowledging and managing the research team’s subjectivity. The team consisted of two co-investigators: Tebogo T. Mamalelala, a PhD-prepared nurse with experience in qualitative research, and Esther I. Ntsayagae, a mental health specialist and PhD-prepared nurse in the Department of Psychiatric Nursing. The principal researcher, Jappy M. Toe, who has extensive training in qualitative research, led the study with the support of trained research assistants involved in data collection. Although the research team did not have a clinical relationship with the participants, they possessed a strong understanding of the broader context of epilepsy care, which informed their insights without introducing bias. To minimise the influence of preconceptions during data collection and analysis, the principal researcher engaged in reflexive journaling, sought regular feedback from supervisors and facilitated peer discussions during the coding and theme development process. These strategies were crucial in ensuring that the findings remained grounded in the participants’ lived experiences while acknowledging the interpretative role of the researcher. Importantly, the researchers had no prior relationships with the participants before recruitment, which allowed for a more objective inquiry.

### Ethical considerations

Permission for the study was granted by the Office of Research and Development (ORD) Institutional Review Board (IRB) at the University of Botswana (UBR/RES/IRB/BIO/GRAD/341) and the Ministry of Health (HPRD:6/14/1). Additionally, approval was obtained from the IRB Ethical Committees at Princess Marina Hospital (PMH 2/11AII [796]). This study adhered to the ethical principles of the Declaration of Helsinki, ensuring informed consent, anonymity, confidentiality and voluntary participation. Written informed consent was secured from all participants prior to study involvement, with the right to withdraw at any time free from coercion or undue influence.

## Results

### Participants’ demographic characteristics

Thirteen caregivers participated in the study. Participants ranged in age from 30 years to 71 years, with caregiving experience spanning 1–41 years. An initial sample of 10 caregivers was expanded to 13 to achieve data saturation. Caregivers were purposively recruited and included mothers, fathers, siblings, spouses and other relatives. Participants represented varied ages, genders, educational levels and employment status, providing a broad range of caregiving perspectives within the Botswana epilepsy context. Demographic characteristics of participants are summarised in Table 1.

**TABLE 1:** Demographic characteristics of participants.

ID	Age (years)	Sex	Relationship	Education level	Marital status	Employment status
P01	48	Female	Mother	Primary	Single	Unemployed
P02	46	Female	Mother	Tertiary	Married	Full-time employed
P03	37	Female	Sister	Tertiary	Married	Full-time employed
P04	48	Male	Father	Secondary	Married	Self-employed
P05	56	Male	Uncle	Secondary	Married	Full-time employed
P06	71	Female	Mother	Tertiary	Single	Retired
P07	38	Male	Husband	Secondary	Married	Full-time employed
P08	52	Female	Mother	Tertiary	Single	Retired
P09	60	Female	Sister	Tertiary	Single	Retired
P10	56	Female	Mother	Primary	Single	Part-time employed
P11	59	Female	Mother	Tertiary	Single	Retired
P12	49	Female	Mother	Secondary	Single	Unemployed
P13	32	Female	Mother	Tertiary	Co-habiting	Self-employed

ID, identification.

### Thematic results

The experiences of caregivers providing long-term care to individuals with epilepsy are shaped by a complex interplay of emotional, physical and social challenges. Several key themes have emerged from their narratives, illuminating the profound impact of caregiving on their well-being. These themes include the emotional burden of fear and anxiety, the physical and mental exhaustion inherent in their roles, the pervasive feelings of depression and helplessness and the stigma and isolation they encounter both in their communities and within their personal lives. Together, these themes paint a vivid picture of the difficulties faced by caregivers, highlighting the need for greater support and understanding of their struggles (see Table 2).

**TABLE 2:** Descriptive coding summary table.

Theme	Sub-theme
1. Emotional burden	1.1. Fear and anxiety 1.2. Physical and mental exhaustion 1.3. Depression and helplessness 1.4. Stigma and isolation
2. Financial burden	2.1. Cost of medication and transportation 2.2. Loss of income
3. Coping mechanism	3.1. Acceptance and patience 3.2. Spiritual support 3.3. Family and social support 3.4. Self-reliance and inner strength

#### Theme 1: Emotional burden

Caregivers reported a substantial emotional burden associated with providing long-term care to individuals with epilepsy. This burden was characterised by persistent psychological distress arising from recurrent seizure episodes, uncertainty regarding disease progression and the need for constant vigilance. Participants described ongoing fear and anxiety, helplessness and emotional exhaustion, reflecting the unpredictable nature of epilepsy and its impact on daily life.

Although caregiving was frequently described as an expression of love and commitment, the cumulative demands often compromised caregivers’ emotional well-being and quality of life.

Concerns about the chronic and lifelong nature of epilepsy further intensified feelings of worry, frustration and emotional fatigue.

**Sub-theme 1.1: Fear and anxiety:** Fear and anxiety were dominant emotions among caregivers. Many participants lived in constant worry about the uncertain onset of seizures, potential injuries and the risk of losing a loved one. This persistent fear shapes their routine and limits their ability to engage in normal activities. Some participants also worried deeply about what would happen to their loved ones if they were no longer able to provide care, particularly as they aged. This fear about the future added another layer of emotional distress and uncertainty. Participants indicated that:

‘Most of the time, I feel very bad for him, because the seizures can be very severe to the point that I can be afraid I might lose him.’ (Participant 03, 37 years, sister)

‘Now, at 15, a young girl should know how to cook and do certain things. I cannot teach her those things; I feel like she would have an attack and get burnt.’ (P04, 46 years, father)

‘The illness just comes unexpectedly, without warning … If he has an attack, I should be there to assist him. If he’s alone, he can even hurt himself.’ (P06, 71 years, mother)

‘What if I am dead? Will it be the same? I am aging, I am 71 years old now.’ (P06, 71 years, mother)

**Sub-theme 1.2: Physical and mental exhaustion:** Caregivers described profound physical and mental exhaustion resulting from continuous caregiving demands and a lack of respite. Many reported that their lives revolved entirely around the person with epilepsy, leaving minimal opportunity for rest or self-care. Constant supervision and physically demanding care tasks contributed to fatigue and burnout:

‘I cannot go anywhere without him, and I cannot leave him here without anybody.’ (P01, 48 years, mother)

‘He cannot do anything for himself … I have to do everything for him.’ (P011, 59 years, mother)

**Sub-theme 1.3: Depression and helplessness:** Feelings of depression and helplessness were frequently reported. Participants described sleep disturbances, persistent sadness and emotional overwhelm, often linked to the perceived inability to alter the course of the illness. Witnessing their loved one’s suffering intensified feelings of despair:

‘It was too heavy for me, I used to cry all night. I was depressed for real, to the extent it affected my work.’ (Participant 03, 37 years, sister)

‘Sometimes I will be thinking about a solution, and no sleep can come to me, sometimes the whole night. Just worrying … It hurts, seeing your only child go through what you cannot explain.’ (P04, 48 years, father)

‘I ended up staying up most of the nights due to the condition. I have insomnia that eventually resulted in depression.’ (P02, 46 years, mother)

**Sub-theme 1.4: Stigma and isolation:** Stigma has become another major challenge for many caregivers. It is often linked to cultural myths and societal misconceptions about epilepsy. Many participants describe their experiences with direct and indirect stigma, which usually results in discrimination and self-isolation for both people living with epilepsy and their caregivers. The community’s negative attitudes, cultural beliefs about the illness and fear of contact with seizures all contribute to the overarching theme.

Some caregivers recounted instances where community members avoided them due to the misconception and fear of epilepsy:

‘“We attended a function, and she had an attack. What I saw shocked me. I thought people were going to rush to her aid, but instead, everyone ran away except me” … and because of that, “she refused to go out or attend any function again”.’ (P07, 38 years, husband)

‘Some people fear people who have epilepsy. Once they have an attack, they run away.’ (P02, 46 years, mother)

Other caregivers provided instances that showed cultural myths delay access to biomedical treatment, attributing epilepsy to demonic or spiritual attacks and suggesting it needs to be treated as such:

‘It is an evil spirit attack, so we should take him to an herbalist.’ (P04, 48 years, father)

Epilepsy is widely believed to be caused by an evil spirit or some punishment from the spirits:

‘In our country, it’s like some evil disease, which has to do with being punished for certain things.’ (P02, 46 years, mother)

‘All you hear in the community, people attribute it to witchcraft.’ (Participant 03, 37 years, sister)

‘They also say that it is associated with a dwarf. I don’t know if it is from a dwarf. Some people would say that the person ate a dwarf, and the spirit entered them.’ (P02, 46 years, mother)

Besides cultural myth and stigma, caregivers are also isolated because of their caregiving roles:

‘Nothing made my role easy. I can’t go anywhere.’ (P01, 48 years, mother)

‘I can’t travel because of him.’ (Participant 03, 37 years, sister)

Stigma and isolation affected the emotional well-being of caregivers and limited potential access to support systems. This theme demonstrates the negative attitude of the community that significantly influences the lived experiences of caregivers. It also closely links to both research questions by highlighting the limitations and psychological challenges they face in building effective support networks.

#### Theme 2: Financial burden

Financial strain emerged as a pervasive challenge across participants’ accounts. Caregiving responsibilities disrupted income-generating activities while simultaneously increasing household expenditures related to medication, transportation and special dietary needs. This economic pressure compounded emotional distress, creating a reciprocal relationship between financial hardship and psychosocial burden.

**Sub-theme 2.1: Cost of medication and transportation:** Medication shortages in public health facilities frequently forced caregivers to purchase antiepileptic drugs from private pharmacies at unaffordable prices. Even caregivers with medical aid reported financial strain due to escalating costs:

‘A box of carbamazepine was going for 800 pula.’ (P02, 46 years, mother)

‘The medication is sometimes unavailable, and we have to buy it at the pharmacy, which can be very expensive.’ (P04, 48 years, father)

Transportation costs further limited access to care, with some caregivers missing appointments due to a lack of funds. Others described borrowing money at high interest rates to travel long distances in search of medication:

‘Sometimes we do not have money for transport.’ (P01, 48 years, mother)

**Sub-theme 2.2: Loss of income:** Many caregivers reported leaving employment or reducing working hours to meet caregiving demands, resulting in long-term financial insecurity:

‘I left work when I was 45 because of this child’s condition. I had to take early retirement.’ (P08, 52 years, mother)

‘I had to stop working … I am just staying at home taking care of him.’ (P01, 48 years, mother)

Additional expenses related to dietary preferences or care needs further strained limited financial resources:

‘Those things he likes are too expensive. I can’t afford them.’ (P01, 48 years, mother)

#### Theme 3: Coping mechanisms

Despite significant challenges, caregivers reported using a range of coping strategies to manage emotional, physical and social stressors. Commonly described strategies included seeking support from family members, engaging in religious or spiritual practices and relying on personal coping mechanisms to continue caregiving responsibilities.

**Sub-theme 3.1: Acceptance and patience:** Acceptance and patience were the most commonly reported coping strategies. Participants described accepting their loved one’s condition as a means of sustaining emotional resilience and continuing caregiving responsibilities:

‘I just accept his condition, that’s all I can do.’ (P01, 48 years, mother)

‘I learned to be patient and to love my son.’ (P04, 48 years, father)

**Sub-theme 3.2: Spiritual support:** Faith and spirituality, particularly prayer, served as important sources of emotional comfort and strength. Caregivers described prayer as a means of coping with uncertainty and restoring hope during periods of distress:

‘Absolutely nothing has made my role easier, except prayers.’ (Participant 03, 37 years, sister)

**Sub-theme 3.3: Family and social support:** Support from spouses and family members helped reduce emotional and physical strain for some caregivers. However, others reported minimal or no support, contributing to isolation and increased distress:

‘My wife … has been my support system.’ (P04, 48 years, father)

‘I did not have anybody beside me who could help.’ (P06, 71 years, mother)

**Sub-theme 3.4: Self-reliance and inner strength:** Some caregivers emphasised self-reliance and inner strength as essential coping resources, particularly among male participants who viewed caregiving as a personal responsibility. Others relied on emotional expression and hope as strategies for endurance:

‘As a man, you got to be strong for your family.’ (P04, 48 years, father)

Caregivers in Gaborone, Botswana, reported ongoing emotional distress, physical exhaustion and financial difficulties related to caring for someone with epilepsy. Coping strategies identified in the study included acceptance, spirituality, family support and self-reliance.

## Discussion

This study explored the lived experiences of caregivers of people with epilepsy in Gaborone, Botswana, with particular attention to emotional burden, financial strain and coping mechanisms. The findings provide context-specific insight into how caregiving is shaped by psychosocial, cultural and structural factors, while also resonating with evidence from other low- and middle-income settings. Overall, the results underscore the multidimensional nature of caregiver burden and highlight the need for integrated, caregiver-centred interventions. These findings extend existing qualitative evidence by illustrating how emotional and financial burdens intersect within a resource-limited epilepsy care context, shaping caregivers’ psychosocial well-being over time.

The predominance of female caregivers in this study reflects entrenched gender norms that position caregiving as a woman’s responsibility. Similar patterns have been reported across African and global contexts, where women disproportionately shoulder unpaid caregiving roles.^32,33,34^ The findings suggest that female caregivers experience compounded stress as they balance caregiving with household responsibilities and, in some cases, employment.^3,35^ This gendered distribution of care highlights caregiving as a socially constructed role shaped by cultural expectations rather than an individual choice.^36,37,38^ The limited representation of male caregivers further suggests that men may engage differently in caregiving or are less likely to be socially assigned such roles, warranting further exploration in future research.

Caregivers in this study experienced substantial emotional burden, characterised by persistent anxiety, fear, psychological distress and exhaustion. The unpredictable nature of seizures and the constant need for vigilance disrupted daily routines and restricted social participation, intensifying emotional strain. These findings are consistent with previous studies reporting heightened anxiety, depression and emotional exhaustion among caregivers of people with epilepsy.^39,40,41,42^

Contrasting evidence from Nepal^43^ suggests that caregiver burden is not uniform and may be moderated by sociocultural support, healthcare access and family structures. Seizure control alone did not predict caregiver burden, reinforcing the notion that psychosocial well-being is influenced by broader contextual and systemic factors.^44^ In the present study, emotional distress appeared closely linked to limited healthcare resources, financial strain and uncertainty about future care arrangements. These findings highlight the importance of addressing caregiving burden through holistic, context-sensitive approaches rather than focusing solely on clinical disease management.^5,44^

Financial burden emerged as a central and pervasive challenge. Caregiving responsibilities frequently disrupted employment, resulting in reduced income or premature withdrawal from the workforce. This economic instability was compounded by out-of-pocket expenditures related to medication, transportation and dietary needs. Similar findings have been reported in South Africa^3^ and in Uganda,^2^ the Democratic Republic of Congo,^4^ and other low-resource settings, where caregivers face systemic barriers such as medication shortages and limited social protection.

The present findings suggest that financial strain not only undermines caregivers’ economic stability, but also exacerbates emotional distress, creating a cyclical relationship between poverty and psychological burden. Vulnerability significantly contributes to caregiver stress, supporting this study findings,^5,44^ who identified economic vulnerability as a key contributor to caregiver stress. Addressing financial hardship is therefore integral to improving caregivers’ psychosocial well-being and capacity to provide sustained care.

Caregivers employed a range of coping strategies to manage caregiving demands, with spirituality and family support emerging as prominent resources. Faith-based practices, particularly prayer, provided emotional comfort, hope and a sense of meaning, enabling caregivers to endure uncertainty and stress. These findings are consistent with previous research demonstrating the protective role of spirituality in reducing psychological distress among caregivers of individuals with chronic illness.^10^

Family support also played a critical role in alleviating emotional and physical strain; however, reliance on informal support systems was often unstable and insufficient. Some caregivers reported isolation due to limited family involvement, highlighting inequities in access to support. Prior studies have similarly shown that perceived social support is associated with reduced depression and anxiety among caregivers of people with epilepsy.^8^ Together, these findings suggest that while informal coping strategies are valuable, they require reinforcement through structured psychological and social support services.^45,46,47^

### Implications

The findings have important implications for nursing practice, education, policy and future research. In practice, caregivers should be recognised as integral partners in epilepsy care. Routine assessment of their psychological well-being, stress levels and support needs should be incorporated in care delivery. Family-inclusive and caregiver-centred care approaches are essential to improving both caregiver and patient outcomes. Nursing education should prepare nurses to identify caregiver distress early, provide education, offer emotional support and make appropriate referrals.

From a policy perspective, integrating epilepsy care into Botswana’s community-based home care services could provide structured support through education, counselling, medication monitoring and stigma reduction. The development of respite care services is particularly critical, as continuous caregiving without relief was a major contributor to emotional and financial strain. These measures aim to enhance support for caregivers and align epilepsy care with a more responsive, caregiver-centred health system.

Future research should employ longitudinal designs with larger and more diverse samples to examine changes in caregiver burden over time and evaluate the effectiveness of targeted interventions. Further exploration of cultural and spiritual coping mechanisms, as well as comparative studies involving caregivers of other neurological and psychiatric conditions, would deepen understanding and inform comprehensive support strategies.

### Study limitations

This study focused on informal, unpaid caregivers and the findings should be interpreted within this context. While caregiving experiences may differ from those of formal, paid caregivers, the results are likely transferable to settings where informal, family-based caregiving predominates.

In particular, the findings apply to similar rural and resource-constrained settings in Botswana, South Africa, and across sub-Saharan Africa, where health system limitations and reliance on family caregivers are common. Shared contextual features such as limited access to healthcare services, fragmented referral systems and the central role of households in supporting care the relevance of these findings beyond the study setting. The provision of detailed contextual descriptions, including caregiving relationships, household structures and health system barriers, further enables readers to assess the applicability of the findings to comparable environments.

However, the sample composition may have limited the breadth of experiences captured. Although participants varied in age, sex and caregiving relationship, the sample was largely composed of family caregivers, particularly mothers. As a result, some perspectives, such as those of male caregivers or less central family members, may have been underrepresented, and certain aspects of caregiving may not have been fully explored. Future research should include more diverse caregiver groups to enhance the transferability of findings across different contexts and populations.

## Conclusion

The findings highlight the profound emotional and financial burden experienced by caregivers of people with epilepsy in Botswana. While caregivers demonstrate resilience through spirituality, acceptance and family support, these strategies alone are insufficient to offset the challenges imposed by systemic and structural constraints. Addressing caregivers’ psychosocial well-being requires integrated interventions that combine psychological support, financial assistance and policy-level reforms to enhance caregivers’ quality of life and sustain long-term epilepsy care. The findings enhance our understanding by emphasising the importance of a caregiver-centred approach in epilepsy care in Botswana.
